# Dr. Randolph’s Case of Nephritic Congestion, with Enormous Enlargement and Softening of the Organ; Produced by Violent Contusion

**Published:** 1842-06-18

**Authors:** Robert C. Randolph

**Affiliations:** Millwood, Va.


MEDICAL EXAMINER.

NEW SERIES.

No. 25.] PHILADELPHIA, JUNE 18, 1842. [Vol. I.

### *Case of Nephritic Congestion, with Enormous Enlargement and Softening of the Organ; produced by violent Contusion.*

By Robert C. Randolph, M. D.

Old Charles, a negro man, belonging to Mr. ———, of Clarke county, Virginia, was sent into the field to drive up a horse on the 1st day of May, 1842. The animal permitted him to halter it in the field, but being very playful, the old man, to prevent its escape, wrapped the chain around his wrist, and secured it, so that it could not slip; in a few minutes the horse attempting to get away pulled him down, and dragged him for some distance over a very rocky surface, bruising him exceedingly, but not wounding him.

He was bled within twenty-four hours, and took some purgative medicine.

May 4th. The above was related to me by Mr. ———, who remarked that he had been passing bloody urine, but was better; he requested me, however, to call and see him, which I did, and found him well enough to rise from his bed and meet me at the door; said he was better, the blood had stopped, but he still felt very sore all around the bottom of his belly. This man had scrotal hernia on the left side, and inguinal on the right. I told him to take a dose of oil, and did not expect to see him again.

6th. He was taken with repeated attacks of approaching syncope, and I was sent for in haste. (Distance five miles.) I found him better; pulse rather quick, and in a *heavy cold sweat*, (as we say in the country;) mental faculties perfectly clear; great tenderness over the whole hypogastric region, and considerable tension; to this locality he referred all his uneasiness. ℞. Bathe abdomen with Spirits of Camphor, and give him small doses of Calomel and Ipecac. at intervals of three or four hours.

7th. Easier; no return of syncope or cold sweats; bowels have been gently moved, but no abatement of tenderness or tension. Tongue dry; pulse quick; surface rather cool than otherwise. ℞. Continue Calomel and Ipecac., with teaspoonful of Spirits of Turpentine every four hours.

8th. Appears better in every respect; scarcely any general constitutional disturbance, yet too much fulness, but very little soreness. ℞. Pulv. Doveri, gr. x. every night, and bowels to be kept open with Castor Oil and Spirits of Turpentine.

12th. Much the same, constitutionally, but considerable increase of fulness and pain in the left hypogastric; the right being free from soreness and softer. ℞. Blister the part affected, give Calomel and Ipecac. powders, morn, noon, and night, to keep the bowels freely open.

14th. Pain relieved by the blister, but there was slight strangury, and urine tinged with blood. ℞. Bowels to be kept open, and give teaspoonful of Balsam Copaiva morn, noon, and night.

16th. Much the same this morning, but last night he had recourse to the blister for relief from violent pain. Strangury continues; no suppression of the secretion, or retention in the bladder, but very high coloured and acrid; thought the blister might have been the cause, and ordered a suspension of its farther use, but continued the balsam.

18th. Matters much in statu quo. ℞. Balsam probably too irritating; discontinue it. Inject the bladder with flaxseed tea, and a little laudanum; keep the bowels open, and use diluents freely.

19th. Tumour much the same; discharge of urine rather increased in quantity and frequency; irritation of the urethra very considerable; proportion of blood about the same. ℞. Continue last prescription, and in addition use infusion of Uva Ursi. The members of the family consider him to be improving, and so would any casual observer, owing to the very slight constitutional disturbance; but since the 14th I have had but little hope of him.

20th. The family still think him improving, but I see very little change, except an increased secretory action, which I do not think desirable. ℞. Stop the Uva Ursi, and give Pulv. Doveri gr. v., Sac. Saturni gr. ij. every six hours; discontinue the injections, but keep up the mucilaginous diluent drinks.

21st. Did not see the patient, but saw his master, who thinks him decidedly better: much less urgency and irritation in the passage of the urine, and not so high coloured.

22d. From the report of yesterday I had some hope of finding my patient improving; my astonishment may be surmised at finding the old man relieved of all his earthly sufferings, and quietly reposing in his winding sheet. I must confess, I was not prepared for this sudden conclusion, for, hopeless as the case had been, I thought it would have lingered longer, and involved the general system in a more decided participation of disease.

I have thus, in a general way, given the most prominent symptoms of the case, which from the first I considered obscure, rendered so chiefly by the absence of pain in the proper region of the kidney, and the exemption from constitutional implication; the digestive and cerebral organs throughout the whole course of the disease being in a healthy condition, the inflammation appearing to expend itself entirely upon its local origin. The treatment I have also given in a loose way, not deeming it of any practical importance. A notice of the morbid condition being my only object, I will proceed to state it, but must first give the account of his death as related to me. He continued apparently better until about noon of the 21st, when the urgency and irritation in the passage of urine suddenly increased, and at last it was entirely suppressed; he died in great pain about 10 o'clock the same evening.

In this explanation I could not find satisfactory cause for so sudden an exit; sufficient time not having elapsed for an over distension of the bladder, or the supervention of peritoneal inflammation, which could run its course to a fatal termination. He was perfectly in his senses up to the time of his death.

Since writing the above I have seen Mr. ———, (the old man's master,) who was present a few minutes previous to his death, and his impression was, that his death was caused by strangulation of the scrotal hernia, from the intensity of pain which he complained of in the inguinal region, up to the moment of his death. None of the ordinary symptoms of strangulation were however presented.

About eighteen hours after his death, my friend Dr. John P. Smith and myself proceeded to make a hurried examination of the body, or rather, of what we considered to be the diseased portion of it.

The first section was made, as for the operation in hernia, on the left side; the sac was found empty, the whole of its contents having receded into the abdominal cavity; the section was then carried on in a direct line upwards and outward until its farther progress was hindered by the ribs; having cut down upon the hernial tumour on the right side, we made a triangular flap of the abdominal wall, by cutting across the recti muscles, &c. &c., just above the symphysis pubis, by lifting which flap the extent of disease was brought into view. Just beneath, and in close contact, throughout the whole length of the first incision, (of course excepting that portion of it extending into the scrotum,) was presented to our view an immense tumour, resembling in colour and consistence the liver, which, upon being cut into, made somewhat the same kind of resistance to the scalpel which the substance of the liver would do; by this I mean, that in cutting, it felt like a firm substance, but as soon as it was exposed to the atmosphere it seemed to melt down into a coagulum, and proceed rapidly into farther decomposition. This was the case with the left half of the organ, in which there appeared to be complete disorganization, whereas, upon making a longitudinal incision in the right side, parallel with the first, the natural structure was not entirely destroyed, but in a state of hypertrophy; and, I doubt not, a partial secretion was carried on to the last, when, finally, the ureter becoming blocked up, it was infiltrated throughout the diseased mass, adding fuel to the existing flame, and hastening the close of an existence which could not long have been protracted by any human agency. We made no accurate measurement of the mass, but it was certainly over one foot in length, and filled up the whole of the left half of the cavity of the abdomen, projecting up under the ribs, and down into the pelvic cavity. The circumference it was impossible to ascertain, as it could not be dissected out entire.

The right kidney was of natural size, and healthy.

The bowels, which were all packed over into the right side of the abdominal cavity, had some appearance of inflammation in different parts.

The liver was very much reduced in size.

The spleen, of natural size and position, but compressed by the distended kidney.

The bladder was not distended, but contained some of the fluid which had been passing off during the course of the disease; we did not examine its coats, nor did we find any lesion of any of the organs or viscera of the abdomen.

I have given this hasty sketch, simply because it was to me an uncommon case, and I thought might be interesting to some of my professional brethren. It will of course be seen that a sufficiently active treatment was not pursued in the commencement of the case. This neglect (if it may be considered such,) will probably be excused by those of the profession who have practised in the country, on that class of our community to which this relates, for reasons unnecessary to mention here; more especially as it will be seen that no alarming symptom occurred until the sixth day after the accident, at which time I thought the course pursued most advisable; nor do I now think that any other would have arrested the progress of so formidable a disease.

The inconvenience in making an autopsy in the country, and the great opposition to them in general, must always render our examinations more cursory than they should be, particularly when not intended for publication, as was the case in this instance.

Should the editors of the Examiner deem this case worthy of publication, it is at their service.

*Millwood, Va., June 6th*, 1842.

